# The College of Physicians of Philadelphia

**Published:** 1899-12

**Authors:** 


### The College of Physicians of Philadelphia.

The next award of the Alvarenga Prize will be made on July 14th, 1900. Essays intended for competition may be upon any subject in medicine, and must be received by the Secretary of the College on or before May 1st, 1900. Further information may be obtained from the Secretary, Dr. Thomas R. Neilson.

